# Radiation Protection in Orthopaedic Surgery: A Regional Survey

**DOI:** 10.7759/cureus.74122

**Published:** 2024-11-20

**Authors:** Dhiraj Sharma, Kate Spacey, Ignatius Liew, Mike Dunne, Vivek Sharma

**Affiliations:** 1 Department of Trauma and Orthopaedics, Addenbrooke's Hospital, Cambridge University Hospitals NHS Foundation Trust, Cambridge, GBR; 2 Department of Trauma and Orthopaedics, Norfolk and Norwich University Hospitals NHS Foundation Trust, Norwich, GBR; 3 Department of Trauma and Orthopaedics, West Norfolk Hospital, Bury St Edmunds, GBR

**Keywords:** ionising radiation exposure, occupational health hazards, orthopaedic surgery, radiation protection, radiation training

## Abstract

Introduction

Orthopaedic surgery frequently involves the use of intra-operative radiographs, commonly taken with surgeons standing in close proximity to the X-ray machine. Radiation training and appropriate radiation protection minimise the harm that surgeons can face from ionising radiation. This study evaluates the current state of radiation training and protective equipment available to orthopaedic surgeons in the East of England.

Methods

A digital questionnaire on Google Forms (Google LLC, Mountain View, CA, USA) was disseminated to regional orthopaedic surgeons between October 2022 and January 2023.

Results

Of the 75 respondents, 49% were consultants and 51% were trainees at various stages. Regarding training and risk assessment, 62% of surgeons lacked radiation protection training, 45% of female trainees didn't undergo radiation risk assessments during pregnancy, and none did while breastfeeding. Concerning radiation protection, all respondents inadvertently X-rayed their hands; lead gloves and glasses were scarce, and X-ray gowns were in poor condition and in a limited range of sizes. Most surgeons were unwilling to purchase their own dosimeters, lead gowns, thyroid guards, and lead glasses, despite their limited availability, inadequate size range, and poor condition. No significant differences were observed between consultant and registrar responses.

Conclusion

The study reveals inadequate training and mitigation against radiation exposure in orthopaedics. The lack of radiation training prompted its inclusion in this year's trainee inductions. The shortage of proper radiation protective equipment exposes surgeons to radiation risks. Orthopaedic surgeons should not have to choose between workplace health and safety and affordability. Greater financial support and investment in radiation protection should be prioritised to minimise radiation-induced harm. We aim to raise awareness and encourage key decision-makers to address this critical issue.

## Introduction

Radiographs play a crucial role in orthopaedic surgery, guiding almost every non-arthroplasty procedure. Surgeons stand in close proximity to scanning equipment, which, along with frequent use, can lead to considerable ionising radiation exposure. Healthcare-related exposure to radiation has been associated with an increased risk of developing radiation-induced malignancies and cataracts [1-3]. Rontgen first reported the discovery of X-rays in 1895, and within a year, the first case report of radiation-induced skin damage was published [4]. Imaging using ionising radiation has since remained indispensable in medicine, but clear data on safe exposure do not exist. Instead, practitioners abide by the ALARA (as low as reasonably achievable) principles as a means of minimising unnecessary radiation exposure [5].

The Health and Safety Executive of the United Kingdom has set guidelines for workplaces where ionising radiation is present [6]. They recommend regular risk assessments, the use of protective equipment such as lead gowns, thyroid guards, lead glasses, and dosimeters, and adequate training to ensure workers' safety. Radiation risk management is the responsibility of the employer in the UK.

Concerns have been raised about the adequacy of radiation protection measures among orthopaedic surgeons, so we designed this study to evaluate the state of training and protection with the aim of highlighting any deficiencies so measures can be taken to improve safety.

This article was previously presented as an oral presentation at the Cambridge Trauma and Orthopaedic Meeting on the 30th of June 2023.

## Materials and methods

Survey contents

A digital questionnaire was developed to investigate the state of radiation training and radiation protection available to orthopaedic surgeons in the East of England. We aimed to ascertain whether surgeons had received radiation training, whether they had access to the required equipment, and whether this equipment met adequate quality standards. Additionally, we aimed to explore the experiences of female trainees who had undergone pregnancy to understand whether appropriate risk assessments and adjustments to their work and equipment had been made. The purpose of this questionnaire is to enable orthopaedic surgeons to provide anonymous feedback so that we can present their collective experiences.

Administration of the survey

A digital questionnaire was created using Google Forms (Google LLC, Mountain View, CA, USA) targeting orthopaedic surgeons (consultants and registrars) in the East of England training region [7]. The survey was distributed between October 2022 and January 2023 to the email addresses of consultants and registrars within the East of England training region. In total, the survey comprised 21 Likert scale questions and one open-ended question at the end, inviting comments.

Google Forms time-stamped data input and transferred responses to a Google Sheets form. Google Sheets was utilised for survey interpretation and graphical illustrations. The survey was considered a service improvement exercise; therefore, the project did not require multi-site ethical, research governance, or audit approval. A copy of the survey can be found in the Appendices section.

## Results

Of the 75 respondents, 37 (49%) were consultants and 38 (51%) were trainees at various stages of their careers.

Training and risk management

Of the respondents, 46 (62%) reported they had not received any radiation training. Lead aprons need appropriate sizing based on body shape, but 93% (70) of trainees said they did not know how to appropriately size lead aprons for themselves. Among female trainees, 83% (5) had not undergone radiation risk assessments during pregnancy, and none did while breastfeeding.

Radiation protection

Thyroid Guards

Thyroid guards were available to 51% (38) of surgeons. The remaining 49% of surgeons said that thyroid guards were either not available or scarcely available. Nine respondents had purchased their own thyroid guard, and 14 respondents would like to buy their own if they were less expensive.

Eye Protection

Ninety-five percent of respondents said that eye protection was not available. Eight surgeons had brought their own, and 19 respondents indicated they would buy their own if they were less expensive.

Lead Gowns

**Figure 1:**
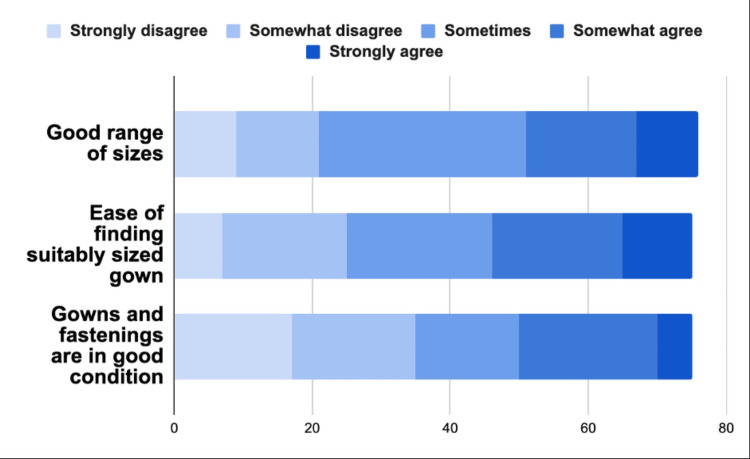
Survey answers on lead gown sizes, availability, and condition

We asked trainees if lead gowns were available in a good range of sizes, whether it was easy to find appropriately sized gowns, and whether the gowns were kept in good condition. Figure 1 demonstrates significant heterogeneity in experiences.

Lead Gloves

Only one respondent said that lead gloves were available. The remaining respondents said they were not sure if they were not available, they were not available, or they were scarcely available. Regarding accidental irradiation of hands, 99% (74) of respondents said they had X-rayed their hands inadvertently during procedures.

Dosimeter

Seventy-three percent (55) of surgeons had never been given a dosimeter during the entirety of their training or consultant careers.

## Discussion

We conducted a survey of orthopaedic surgeons in one of England's largest training deaneries to gain insight into their experiences.

It remains unclear how much radiation is considered 'safe’ because it is not pragmatic to conduct research that can clearly correlate radiation dose with risk. Much of the available guidance is based on a small number of real-world radiation incidents, such as the Life Span Study that followed individuals exposed to the atomic bombs dropped on Hiroshima and Nagasaki [8]. This study demonstrated that higher doses and increased frequency of exposure to ionising radiation increase the risk of cell damage. As a result, the ALARA principles are applied wherever there is a risk of radiation exposure. The principle accepts that low-dose radiation exposure is inevitable, but we should do as much as we can to minimise exposure to reduce the unknown risk.

Training and risk management

Our study findings highlight a worrying gap in the provision of radiation protection measures for orthopaedic surgeons. With the majority of surgeons reporting a lack of radiation protection training, there is a pressing need for this crucial aspect of safety to be incorporated into trainee induction programmes. A study by Saroki et al. previously highlighted the need for such training and recommended it as a mandatory part of the curriculum for medical students and residents [9].

For female surgeons who are pregnant or post-partum, it is important that they undergo radiation risk assessments to ensure that appropriately fitted gowns are provided to protect the foetus and account for the increase in breast tissue mass seen in pregnancy. There is an increased risk of breast cancer in orthopaedic surgeons because breast tissue extends into the axilla - an area not covered by all lead gowns. One study found a 2.9-3.9-fold increased incidence of breast cancer in female orthopaedic surgeons compared to those in the fields of plastic surgery and urology [1,10-12]. Unfortunately, these risks do not appear to have been mitigated among female respondents in this survey.

Radiation protection

This survey highlights the scarcity and poor condition of protective equipment available to orthopaedic surgeons. Low-dose ionising radiation exposure to the hands, breasts, neck, and eyes poses a risk of stochastic damage, leading to neoplasms and cataract formation, and research suggests that healthcare workers are more likely to suffer these consequences [2,10,13]. Unfortunately, there is very little research on the low-level ionising radiation exposure seen in orthopaedic surgery.

The survey highlighted both a lack of availability and poor upkeep of protective equipment, with almost half of respondents not having access to thyroid guards. Surgeons should have access to the correct equipment, which should also be well-maintained and hygienic. The survey demonstrated clear heterogeneity in the quality and upkeep of available protective equipment. Hygienic, well-fitted protective equipment reduces the barrier to surgeons being appropriately protected. A comment in the open-ended survey question made a pragmatic suggestion that disposable thyroid guard covers could help improve cleanliness because, unlike gowns, thyroid guards are worn on the naked neck. This is a reasonable suggestion that might improve the use of thyroid shields but is pointless for those who do not have thyroid guards available in their workplace.

The International Atomic Energy Agency suggests that the risk of orthopaedic surgeons developing radiation-induced malignancies or cataracts is very low if they use the correct protective equipment [14]. Cuenca et al. demonstrated that surgeons receive ocular radiation below safe thresholds, but it is not clear exactly what is proven to be safe after repeated exposure [15]. This is reassuring, but the absence of self-reported consequences does not mean they are not occurring. Cataracts and cancer occur in the normal population, so small increases can be difficult to detect. In cases where there is an unknown effect, it would be reasonable to follow the ALARA principles. In practice, this would involve ensuring that all surgeons have access to full protective equipment. All practitioners strive to obey ALARA principles, but imperfect practice is a reality, as neatly demonstrated by the fact that 99% of respondents had accidentally X-rayed their hands.

Suggestions

We recommend the following steps to enhance radiation protection in orthopaedics. First, provide radiation training to every orthopaedic surgeon at the start of each new placement, with an option to opt out if desired. Second, ensure full-body and eye protection is available to all orthopaedic surgeons, with proper fitting checks conducted at the beginning of every placement and during pregnancy. Third, establish clear national orthopaedic guidelines that can be audited and used as a catalyst for change. Finally, conduct a survey of orthopaedic surgeons across the UK to assess whether there is an increased incidence of cataracts within the profession.

Limitations

This survey only questioned surgeons in one training region in the UK but highlights the need for a national survey to study variation in occupational risk, training, funding of equipment, auditing equipment, and provision of funds for personalised equipment such as thyroid guards and eye protection for those with common visual impairments. The survey predominantly uses Likert-style questions, which provide binary data used to draw conclusions but offer less detail on personalised experiences.

## Conclusions

Our survey highlights significant inadequacies in radiation protection measures and training among orthopaedic surgeons. It is unacceptable that surgeons are forced to choose between their health and safety and the affordability of protective equipment. This study aims to raise awareness about this issue and implore key decision-makers to prioritise investment in radiation protection to minimise potential harm caused by radiation exposure. The inclusion of radiation training in this year's trainee inductions is a step in the right direction, and further improvements must be made to safeguard our healthcare professionals.

## Figures and Tables

**Figure 2:**
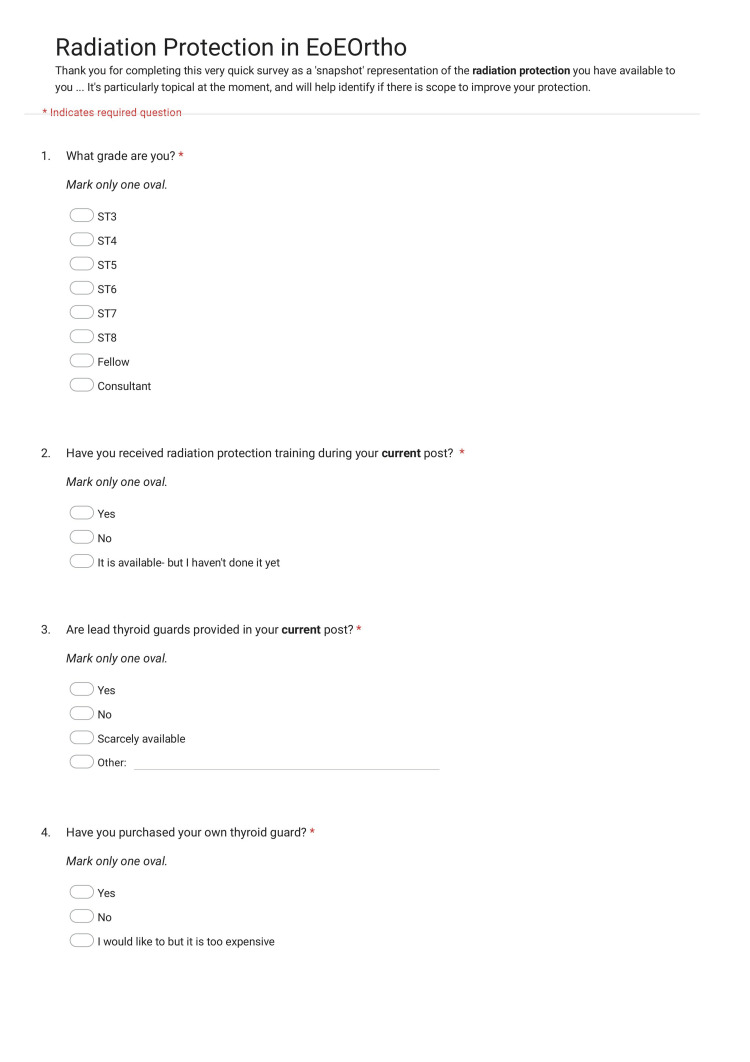
Online survey on radiation training and protection in orthopaedic surgery (page one)

**Figure 3:**
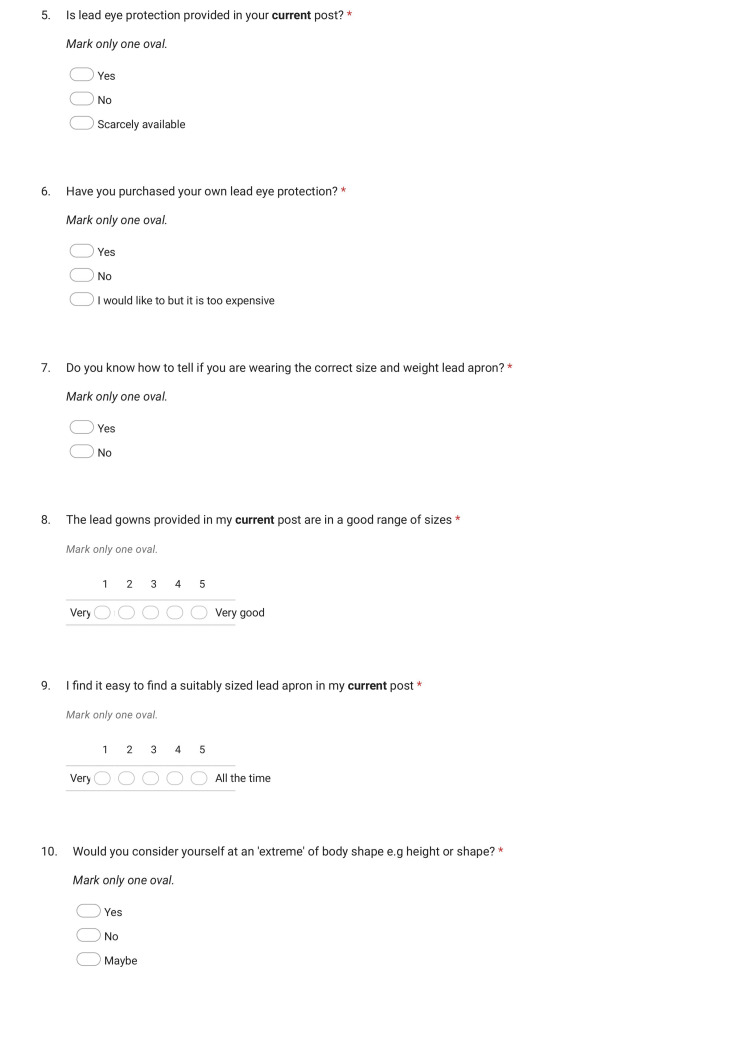
Online survey on radiation training and protection in orthopaedic surgery (page two)

**Figure 4:**
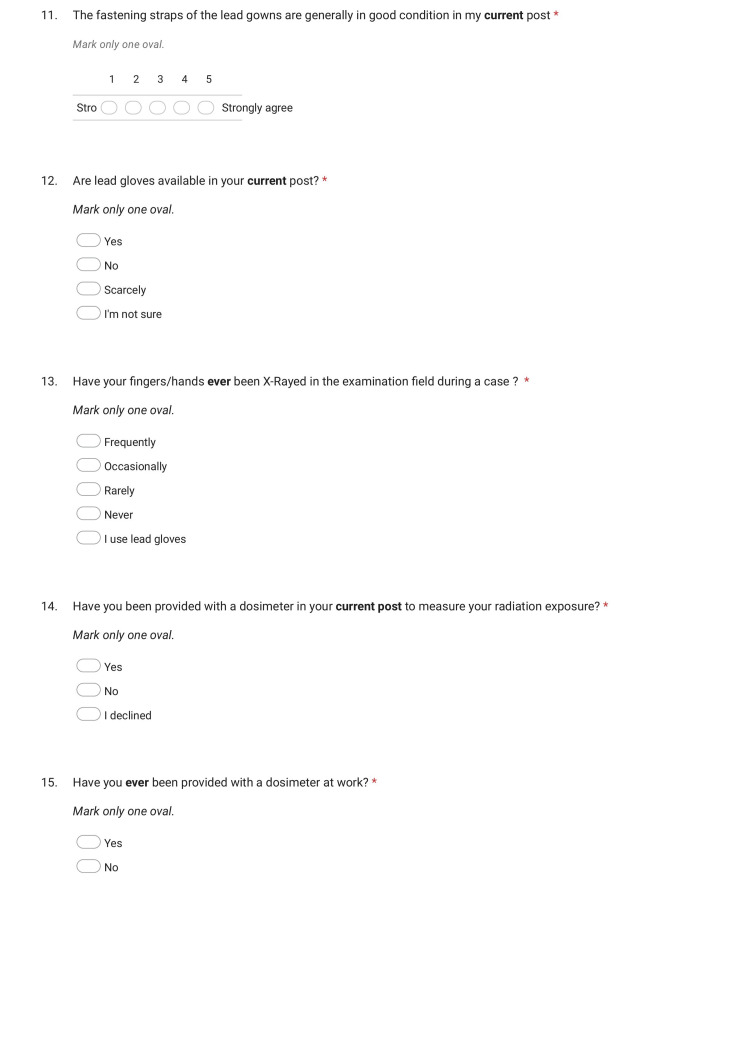
Online survey on radiation training and protection in orthopaedic surgery (page three)

**Figure 5:**
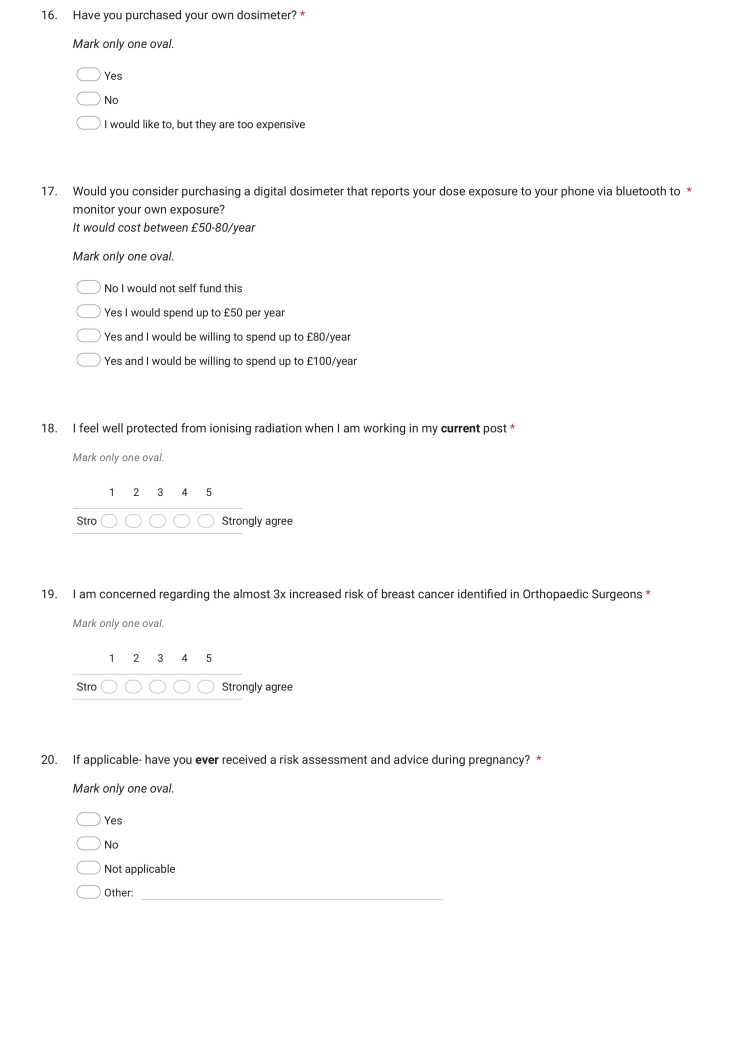
Online survey on radiation training and protection in orthopaedic surgery (page four)

**Figure 6:**
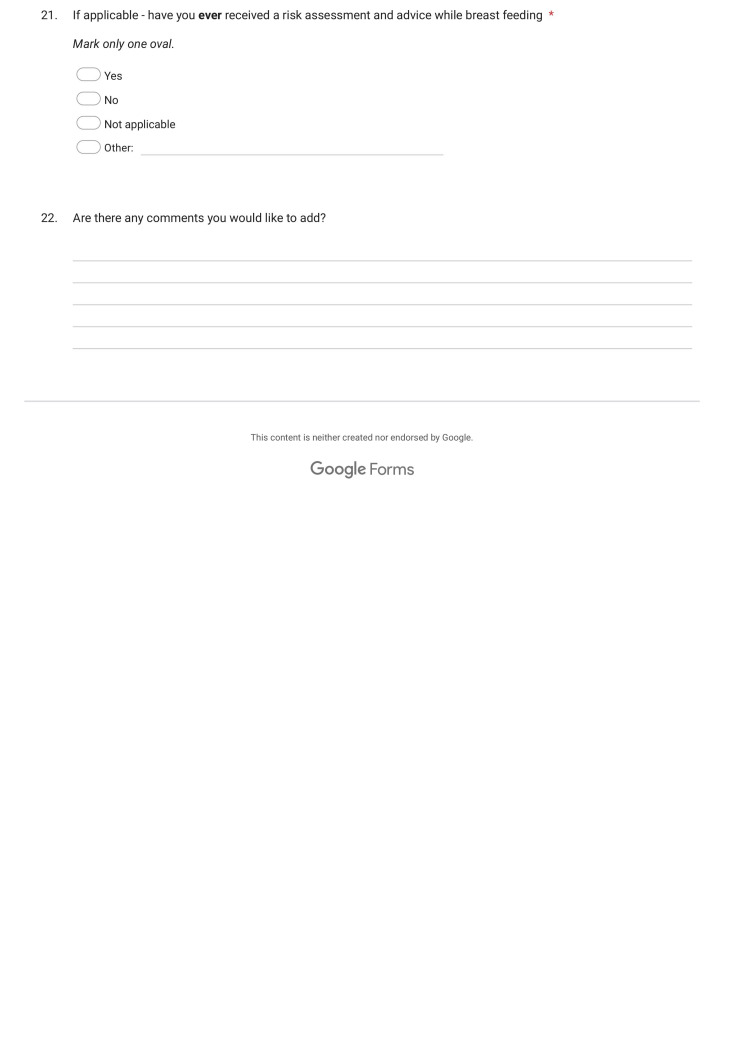
Online survey on radiation training and protection in orthopaedic surgery (page five)
